# Degenerative Changes in Aging Human Pelvic Lymph Nodes—A Reason to Rethink Staging and Therapy of Regional Malignancies?

**DOI:** 10.3390/cancers15194754

**Published:** 2023-09-27

**Authors:** Daniel Gödde, Stephan Degener, Christine Walles, Rosalie Keller, Katharina Graf, Marco Tosch, Susanne Krege, Michael Musch, Hans Michael Kvasnicka, Maximilian Ackermann, Stephan Störkel, Friedrich-Carl von Rundstedt

**Affiliations:** 1 Department of Pathology and Molecular Pathology, Helios University Hospital Wuppertal, 42283 Wuppertal, Germany; 2 Department of Medicine, Faculty of Health, Witten/Herdecke University, 58448 Witten, Germany; 3 Department of Urology, Helios University Hospital Wuppertal, 42283 Wuppertal, Germany; 4 Clinic for Otolaryngology, HELIOS-Hospital, 47805 Krefeld, Germany; 5 Centre for Clinical Trials, Witten/Herdecke University, 58448 Witten, Germany; 6 Department of Nuclear Medicine, Helios University Hospital Wuppertal, 42283 Wuppertal, Germany; 7 Department of Urology, Pediatric Urology and Urologic Oncology, Evangelische Kliniken Essen-Mitte, 45136 Essen, Germany

**Keywords:** pelvic lymph node, immunosenescence, aging, skip metastasis, pelvic urologic carcinoma, bladder cancer, prostate cancer

## Abstract

**Simple Summary:**

Therapy and outcome of urological tumors of the small pelvis depend on preoperative staging and surgical results. In this context, the lymph node status plays a significant role. Lymphogenic metastasis of urological tumors is not fully understood to date. Age-dependent changes in lymph node architecture have not been fully explored, even though they have an impact on lymph node function and therefore on tumor metastatic behavior. In this study, it was shown that the pelvic lymph nodes show more degenerative changes with age, such as increased lipomatous atrophy, calcifications, framework and capsular fibrosis. In addition, a smaller lymph node diameter was observed more frequently with increasing age. The authors believe that a better understanding of age-related lymph node changes can contribute to increasing the quality of staging and therapy.

**Abstract:**

Lymph node metastases are common in pelvic urological tumors, and the age-related remodeling process of the pelvic lymph nodes influences metastatic behavior. The aim of this work is to characterize age-related degenerative changes in the pelvic lymph nodes with respect to their occurrence and extent. A total of 5173 pelvic lymph nodes of 390 patients aged 44 to 79 years (median 68 years, IQR 62–71 years) were histologically examined for degenerative structural changes. Lymph node size, lipomatous atrophy, capsular fibrosis, framework fibrosis, and calcifications were recorded semi-quantitatively and evaluated by age group. Significantly more lymph nodes <10 mm were found in older patients (*p* = 0.001). The incidence of framework fibrosis, capsular fibrosis, and calcifications increased significantly with increasing patient age (*p* < 0.001). In lipomatous atrophy, an increase in mild to moderate lipomatous atrophy was observed with increasing age (*p* < 0.001). In this, the largest study to date on this topic, age-related degenerative changes in pelvic lymph nodes were proven. Due to the consecutive decrease in hte filtration function of pelvic lymph nodes with increasing age, staging and therapy of metastatic pelvic urologic carcinomas should be reconsidered.

## 1. Introduction

Histological examination of bilateral pelvic lymphadenectomy provides the most accurate staging of malignant urological tumors. Furthermore, the pathological findings contribute to the decision on adjuvant therapy [1,2,3,4]. In addition to the determination of tumor extent, in prostate cancer (PC), removal of the pelvic lymph nodes (LN) reduces disease burden and has a significantly positive effect on cancer-specific survival [5,6]. In bladder cancer (BC), predictions of recurrence-free and disease-specific survival can be made based on pN stage, number of LNs involved, number of LNs removed, and LN density [7,8,9]. LN status and the number of LNs affected also determine the outcome of patients with BC treated surgically with a cystectomy [9]. For urological tumors of the pelvis, a lymphadenectomy is recommended from a certain stage, which includes LNs of the obturator, external iliac, and hypogastric areas with or without presacral and common iliac LNs [10,11,12]. The incidence of LN metastases in PC is variably reported in the literature, depending on the study type, and is currently <10% [13,14,15]. With a frequency of approximately 25% at the time of cystectomy, regionary pelvic LN metastases are common in BC [16,17,18]. In this setting, the incidence of LN metastases and tumor spread to more proximal LN sites increases with locally advanced disease/tumor depth (pT) [16,17,19]. Discontinuous spread of tumor cells to LN, so-called skip metastases, occur in BC and in PC [14,17,20,21,22,23]. To date, the occurrence of skip metastasis has been explained by direct lymphatic drainage to proximal, e.g., presacral or aortic, sites [16] or labeling errors [23]. However, there is also evidence that reduced filtering function through structural remodeling of LN enables the spread of microorganisms and tumor cells [24,25,26,27].

Due to increasing age, a complex remodeling of the immune system takes place, called immunosenescence, which also affects LN morphology [28]. LNs have a complex architecture at the cellular level, the basic functional unit of which is the lymphoid lobule [29]. It is worth noting that all structural parts of the LN are subject to age-related changes [30]. The altered microenvironment of the LN contributes significantly to the age-related decline of LN function through disorganization and dysfunction of LN architecture [26,31,32]. Consequently, age-related structural changes in the pelvic LN also have implications for tumor staging and, ultimately, patient care. The aim of this work is to characterize age-related degenerative changes in the pelvic LN in their occurrence and extent.

## 2. Materials and Methods

This retrospective single-center study was conducted in accordance with the ethical principles of the Declaration of Helsinki. Ethics approval was received from the Ethics Committee of the Witten/Herdecke University (Approval No. 20/2016).

**Figure 1:**
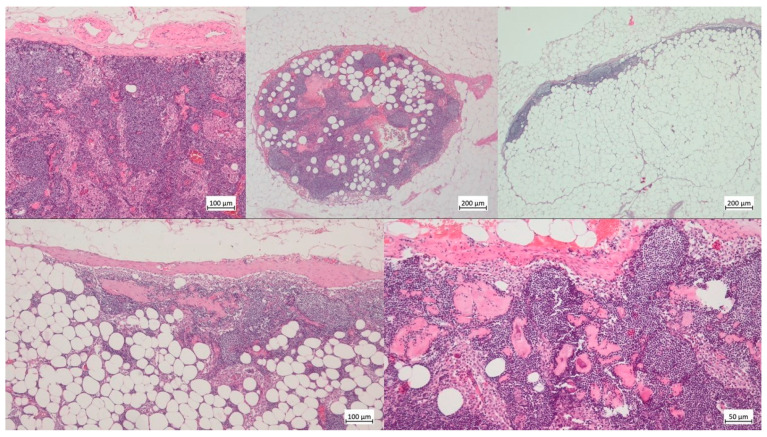
Upper row left: LN with capsular fibrosis (≤30%, H&E, 50×); middle: LN with low framework fibrosis and moderate lipomatous atrophy (31–60%, H&E, 25×); right: LN with subtotal lipomatous atrophy (≥61%, H&E, 25×). Lower row left: LN with low framework fibrosis, capsular fibrosis and subtotal lipomatous atrophy (≥61%, H&E, 50×); right: LN with moderate framework fibrosis and calcifications (H&E, 100×).

The whole resected pelvic fat specimens (stored in 4% buffered formalin) of 390 patients, aged 44 to 79 years (median 68 years, IQR 62–71 years), treated with prostatectomy and extended pelvic lymph node dissection (PLND) for prostate cancer, was embedded for histological evaluation to identify all resected LNs. Dissection was performed manually without the use of a chemical revealing solution. Each lymph node found was lamellated and embedded according to its largest diameter. Complete histological examination on hematoxylin and eosin (H&E) stained step sections with analysis of a complete cross-section was possible in 5173 of 6026 LNs. Lymph node metastasis was ruled out clinically before surgery, while pathological examination found a total of 81 lymph node metastases (1.6%) in 35 patients. Extracapsular growth was detected in 6 lymph node metastases, and was not considered in the survey of lymph node diameter. Structural changes such as lipomatous atrophy, capsular or framework fibrosis, and calcifications were documented as morphological correlates of degenerative changes (Figure 1). Lipomatous atrophy was semi-quantitatively documented as a percentage of the incised area in stages of ≤30%, 31–60%, and ≥61%. Changes in framework fibrosis were recorded as absent, low, moderate, or severe. Capsular fibrosis and calcifications were classified as detectable or undetectable. Furthermore, the LN size was documented. To achieve our goal, the extent of structural change and LN size were compared in three different age groups. The variables were described using absolute number and percentages. Statistical analysis was performed using the chi-square test where necessary. The results are reported as statistically significant whenever *p* < 0.05. The data generated were analyzed using the Statistics Package for Social Sciences version 25 (SPSS, IMB Corp, Armonk, NY, USA).

## 3. Results

The numbers of LNs removed from the right and left side of the body were almost identical (right 3004, 49.9%; left 3022, 51.1%). Most LNs were obtained from the obturator fossa (right 1111, 18.4%; left 1078, 17.9%), followed by LNs from the iliac vasae (externa right 766, 12.7%; externa left 830, 13.8%; interna right 764, 12.7%; interna left 801, 13.3%). Another 676 LNs (11.2%) could only be assigned to the right or left side as the sampling location. Patients and LNs showed an uneven distribution across the three age groups (Table 1).

The pelvic LNs exhibited a follicular structure. Structural changes in LN architecture known from the literature are noted to varying degrees in all examined LNs (Table 2). Overall, 2701 LNs (52.2%, median 5 mm, IQR 3–7 mm) were smaller than 10 mm (<10 mm), while 2472 (47.8%, median 17 mm, IQR 13–24 mm) had a minimum diameter of 10 mm or more (≥10 mm). When comparing the three age groups, significantly more LN < 10 mm were found in older patients, and vice versa (*p* = 0.001). Comparing the different sampling localizations in detail, a more mixed picture emerged. Only the LNs of the vasae iliac interna continuously become smaller with increasing age, while those from other localizations exhibited fluctuations.

While the detailed examination of the sampling locations for framework fibrosis also showed an inconsistent picture, framework fibrosis across all LNs increased significantly with age (*p* < 0.001). This was observed especially in the case of mild structural changes, where the number of LNs without framework fibrosis decreased and the number of LNs with mild framework sclerosis increased with increasing age. Overall, most LNs showed little (23.7%) or no evidence (69.9%) of framework fibrosis (Table 2).

Capsular fibrosis was more common in combination with higher grades of framework fibrosis, and was detected in 1814 LNs (35.1%). A significant increase in capsular fibrosis across all three age groups (*p* < 0.001) was detected (Table 2).

The association with framework fibrosis was even more pronounced for calcifications. A total of 45.9% of the LNs showed calcifications. Here, an increase in LNs with calcifications was observed across all age groups (*p* < 0.001) (Table 2).

Almost all LNs from all anatomic localizations showed lipomatous atrophy to varying degrees. LNs with every quantity of lipomatous atrophy were found in all sizes. An increase in mild to moderate lipomatous atrophy was observed, albeit inconsistently, with increasing age (*p* < 0.001). Only LN with a lipomatous remodeling above ≥61% occurred less with increasing age (Table 2).

## 4. Discussion

LNs are subject to constant morphological changes. As far as is known, the morphological remodeling is mainly age related [33]. Pan et al. postulated a life cycle of generation and degeneration of LNs [30]. Until adolescence, there is a steady increase in lymphoid tissue, whereas regression begins at puberty and continues at a slower rate beyond 40 years of age [34]. With advancing age, adults have less lymphoid tissue in both the cortex and the medulla of LNs [35]. The number and size of germinal centers show the most impressive age-related changes, regardless of their location [36,37]. However, the degenerative changes affect all structural elements of the LN, with areas colonized by various immune cells replaced by connective tissue [30,33,35]. Varieties of degenerative changes have been reported in senescent LNs such as hyalinization, fibrosis, or lipomatous atrophy (Table 3). These structural changes are heterogeneous, variable, continuous, and affect each LN individually [33]. The extent of LN remodeling depends largely on the localization—central LNs with increased antigen stimulation show less remodeling than peripheral LNs, e.g., in the pelvis [35,36]. Furthermore, the nature of the connective tissue filling the increasing cavity of the LN varies with the nutritional state [34]. Degeneration continues until an LN becomes inactive, without lymphatic tissue [30] and with a macroscopic transparent appearance [35]. However, age-related morphological changes are also detectable in lymphatic vessels [38].

In this, the largest collective studied to date, age-related degenerative changes in the LNs of the pelvis were evident and were evaluated semi-quantitatively for the first time. Through the complete investigation of an entire LN site, age-dependent remodeling processes could be depicted in detail. For example, a significant age-related increase in fibrosis of the meshwork and capsule was demonstrated in this collective. The broad spectrum of observed morphological changes is probably a result of the complex microenvironment of the LN site. Due to the different lymphatic influx to the individual LNs, they are stimulated to different extents, and therefore show different degrees of advanced degenerative changes. This hypothesis is consistent with the literature data available to date.

### 4.1. Size

The present data show an average reduction in LN size with increasing age, especially between the 6th and 7th decades of life. The reduction in LN size with increasing age has so far been reported differently in the literature. While some human [30,33,39] as well as animal [40,41,42] studies have described a reduction in LN size partly depending on the localization, others found no significant decreases in LN size [26]. In addition, reductions in the number of LNs are associated with senescence [35]. The individual but ubiquitous LN degeneration can be used as an explanatory factor for these observations. In this context, LN size depends on the degenerative changes described previously, in particular compensating for the loss of lymphoid tissue by lipomatous atrophy. This has already been described as a physiological process in superficial LN with reduced immunological activation [36]. Furthermore, the loss of lymphoid tissue in senescent LNs can also produce cystic spaces containing fluid [43,44]. Conversely, macroscopic identification and measurement of LNs with extensive lipomatous atrophy is challenging. These inactive and “transparent” LNs occur in different sizes and shapes [30], and it is, therefore, conceivable that such LNs may be missed during preparation. The decreasing number of LNs with increasing age can thus be explained by degenerative remodeling processes.

### 4.2. Lipomatous Atrophy

The prevalence and severity of lipomatous atrophy in human LNs appears to increase with senescence [35]. It occurs to varying degrees depending on the nutritional state and the location of the LN, with inguinal, popliteal, cubital, and axillary LNs being more severely affected than mesenteric and deep cervical LNs [34,35]. Lipomatous atrophy can only be seen in almost 30% of mesocolic LNs [45]. Thus, replacement of lymphatic parenchyma by adipose tissue has been described as a feature of peripheral and usually less antigen stimulated LNs [36]. These observations are consistent with the findings collected in this series. As the only structural change in the studied LN morphology, no clear positive correlation with advancing age could be demonstrated for lipomatous atrophy. Only an increase in mild to moderate lipomatous atrophy was observed, albeit inconsistently, with increasing age. This may be explained by variable degenerative changes caused by a variable microenvironment in the pelvic LNs. Alternatively, a non-age-related, i.e., mechanical, process for lipomatous atrophy in pelvic LNs should be considered, so that maximum lipomatous atrophy, which cannot be further increased with age, is reached early.

### 4.3. Hyalinization/Fibrosis of Framework and Capsula

The delicate, porous, sponge-like reticular meshwork of a lymphoid lobule is composed of a cellular and an extracellular component that includes reticular fibers, related extracellular matrix components, and fibroblastic reticular cells (FRCs) [29,46]. Reticular fibers, in addition to other components such as elastin, fibronectin, or laminin-1, consist of a core of collagen fibrils including collagens type I, III and IV, enveloped in a layer of basement membrane [46,47,48]. The increased production of extracellular matrix by FRCs leads to fibrosis of the reticular fibers with remodeling of the microarchitecture of LNs [49,50,51]. Fibrous change does not occur until the medullary lymphocytes have been greatly reduced in number and the reticulum fibers have been closely compacted [34]. Histologically, general fibrosis with enlargement of the capsule and trabeculae and increased content of connective tissue around blood vessels have been observed in the mesenteric LNs of elderly patients [52]. Moreover, dense bundles of collagen fibers have been observed in the marginal sinuses of aging LNs, while the reticular mesh of the sinuses is preserved. Connective tissue fibrosis, as well as the destruction of the reticular stroma, results in an impairment of lymph filtration [53,54,55]. Besides fibrosis, the amount of hyaline deposits increases significantly with advancing age [56]. In hyalinization, differences occur with respect to morphology, proportional volume, and incidence, as well as ethnicity [57]. Since the fibrous capsule is directly connected to the connective tissue of the hilus [34], it can be assumed that the remodeling process also extends to the capsule. However, for other exogenous factors, an influence especially on the fibrosis of LN tissue could be demonstrated. Chronic inflammation contributes to fibrosis in regional LNs by means of an increased number of proinflammatory cytokines influencing the FRCs [35,58,59]. Anthracosis also seems to have an influence on the pathogenesis of hyalinization [57]. In their large study of 2250 axillary LNs harvested postmortem, Tsakraklides et al. demonstrated that fibrosis and hyaline deposits in LNs occurred particularly in chronic disease, and especially in cancer [56]. At first glance, these data contradict the observations made here. However, it can be assumed that two etiologies for fibrotic remodeling of the LNs, one neoplastic and one age related, were observed, since firstly, the patient population of Tsakraklides et al. was younger, and secondly, our observations, exclusively in tumor patients, demonstrated significant age-related effects. To this the thesis fits, that mild fibrosis of inguinal LNs could also be observed in young adults [33], and in starvation, cystic remodeling of the LN hilum is also observed [34].

### 4.4. Calcifications

There are different theories regarding the pathogenesis of calcifications, which have most frequently been described in the context of various inflammatory or neoplastic diseases [60,61,62,63]. As a result of local tissue damage leading to the release of alkaline phosphatase, the local pH of the tissue increases, leading to precipitation of calcium salts [64]. In addition, there are data indicating that calcifications of blood vessels can also occur as a result of the aging process [65,66,67]. As shown here, calcifications of the reticular meshwork of pelvic LN show a relation to increasing age. Since all patients had a tumor, the increase in calcifications cannot be explained by the neoplastic underlying disease alone.

### 4.5. Stromal Niche

The basic functional unit of an LN is the lymphoid lobule, a complex structure which is composed of different cell types [29]. Stromal cells not only form the LN architecture, they also play a role in the development, maintenance, and proliferation of immune cells, as well as in innate and adaptive immune responses [26,32]. Furthermore, intercellular communication also exists between metastatic prostate cancer cells and the fibroblastic microenvironment [68]. FRCs, the largest subpopulation of stromal cells, form a three-dimensional network serving as a scaffold for the movement of lymphocytes [26]. Furthermore, FRCs are the main source of collagen in LNs, an essential component of the extracellular matrix [69]. These collagen fibers determine the proper architecture, and therefore the function, of LNs. However, increased collagen fiber mass is frequently observed in some aging organs, and is associated with fibrosis [58]. The changed microenvironment contributes significantly to the age-associated decline of immune function by disorganization and dysfunction of the LN architecture [26,31,32].

As a result of remodeling processes, gaps form in the superficial cortex, affecting the nodal filtration properties [70]. These gaps could be seen in the abdominal and pelvic LNs of aged Japanese people [43]. In an animal model in rats, the gaps in the meshwork architecture resulted in a rapid shunt for lymphatic flow [71]. An intranodal shunt for lymphatic flow also appears to be common in visceral human LNs in the elderly [70]. Age-related remodeling of LNs with gap formation, and increased shunt flow also impedes the settlement of carcinoma cells in pelvic LNs. Consequently, as shown for prostate carcinoma, metastases occur more frequently in less degeneratively altered LNs [27]. In addition to the complex lymphatic drainage of the pelvic organs [16,72,73], this is a further explanation for the occurrence of skip metastases. There are already initial clinical data identifying age as a risk factor for the occurrence of skip metastases [74,75,76]. To date, the occurrence of skip metastasis in BC has been explained by a direct lymphatic drainage to the proximal, e.g., presacral or aortic, sites or labeling errors [16,17,19,21,23,77]. Moreover, atypical metastatic patterns and the sometimes-wide metastatic extension of urologic tumors of the pelvis may be explained by age-related LN degeneration. Anatomical and radiological studies have demonstrated the complex lymphatic drainage pattern of the bladder and prostate [16,72,73]. All bladder sites have lymphatic drainage to different areas of the pelvis (internal/external/common iliac region, obturator fossa) [72]. The primary lymphatic drainage sites are the external and internal iliac, as well as the obturator region. Secondary lymphatic drainage occurs into the common iliac region up until the uretero–iliac crossing. Lymphatic drainage of the trigone and posterior wall leads directly into the presacral LNs [16,72]. The main sites for LN metastasis in BC are the obturator/hypogastric and external iliac LNs [19]. However, LN metastasis can be found in the common iliac and presacral LNs, as well as in LNs above the aortic bifurcation and the inferior mesenteric artery [16,19]. Moreover, metastases occur in BC in up to 40% in contralateral LNs. The lymphatics of the prostate gland drain primarily into the obturator and iliac LNs, but also into the presacral LNs [72]. In PC, lymphatic spread ascends from the pelvis up to the retroperitoneum, invariably through the common iliac LNs [11].

Because LN metastases are associated with an increased risk of local recurrence and disease progression [16], in addition to staging, reduction of disease burden by lymphadenectomy is critical for patients. The LN status and the number of involved LNs determine the outcome of patients with BC treated with cystectomy [9]. In BC, lymphadenectomy prolongs time to disease recurrence and cancer-specific survival [7]. In PC, lymphadenectomy shows a significant advantage in tumor-specific survival for advanced stages of disease [5,6]. Thus, the goal of surgical tumor therapy must be the complete removal of the main tumor, as well as any regional and distant LN metastases.

**Figure 2:**
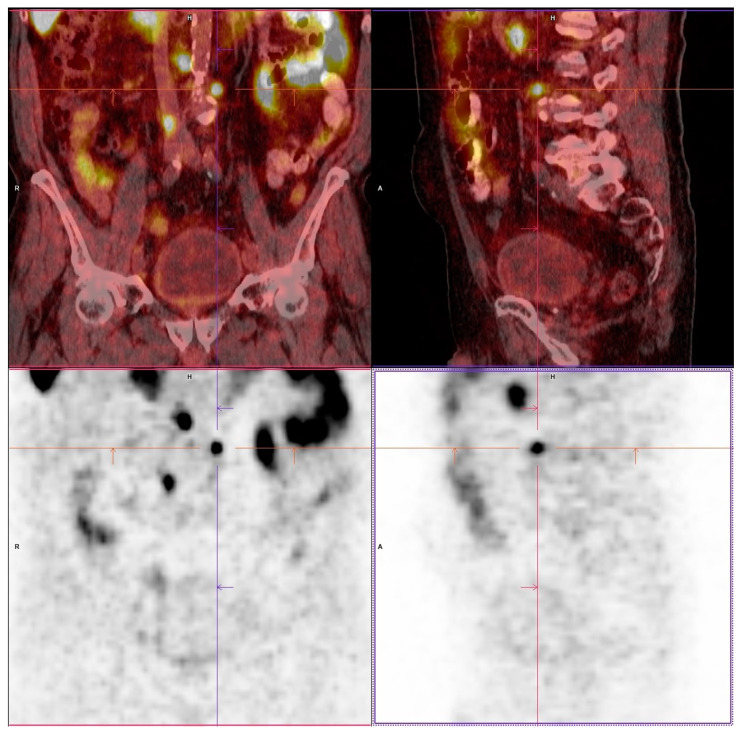
F-18-PSMA-1007 imaging demonstrating a paraaortic metastatic suspicious finding 1 year after local therapy of the primary tumor and increasing PSA-level. Top with combined coronal and sagital CT and PET images, bottom with coronal and sagital PET images.

Overall, it can be postulated that metastasis of carcinoma cells to pelvic LNs depends on tumor biology, lymphatic drainage, and LN morphology. Metastases more often pass through degenerative remodeled LNs due to intranodal shunt formation [70] and settle in less degeneratively altered LN [27]. Considering the age-related morphological changes in the pelvic LNs demonstrated here, a very accurate fat tissue resection of each pelvic lymph node station must be performed to cover all lymph nodes. Otherwise, alternative preoperative examination methods should be considered in the staging process of elderly patients. Prostate-specific membrane antigen (PSMA) imaging can also be used to detect metastases outside of the predefined surgical region, e.g., in ePLND prior to surgery (Figure 2).

Several limitations of this observational retrospective study should be considered. First, our study includes only hospitalized men with ongoing PC. Women in general and non-hospitalized men were not examined in this study. Second, the effects of racial, genetic, hormonal, lifestyle, socioeconomic, and environmental factors on degenerative changes in LN are not captured here.

## 5. Conclusions

The ubiquitous and steady, but not uniform, process of age-related changes was demonstrated in pelvic LNs in the largest study to date on this topic. Due to the increasing degeneration of LNs with old age and their consequently reduced filtration function, an accurate pelvic fat tissue resection is mandatory. Alternatively, the staging of metastatic urologic carcinomas should be reconsidered. To cover all metastases of advanced age during surgical therapy, either the surgical area must be broadly defined or the preoperative use of screening methods (e.g., PSMA-PET in prostate cancer) must be considered.

## Figures and Tables

**Table 1:** Distribution of patients and LNs according to age group.

Age	Number of Patients		Examined LNs	
≤60 y	72	18.50%	927	17.90%
61–70 y	201	51.50%	2668	51.60%
≥71 y	117	30.00%	1578	30.50%
Total	390	100%	5173	100%

**Table 2:** Occurrence and distribution of structural changes in the pelvic LN according to age groups (* significant value indicates differences in age groups).

Structural Change		Over All	≤60 Years	61–70 Years	≥71 Years	*p*
LN size	<10 mm	2701 (52.2%)	458 (49.4%)	1358 (50.9%)	885 (56.1%)	
	≥10 mm	2472 (47.8%)	469 (50.6%)	1310 (49.1%)	693 (43.9%)	0.001 *
Framework fibrosis	none	3614 (69.9%)	726 (78.3%)	1836 (68.8%)	1052(66.7%)	
	milde	1224 (23.7%)	154 (16.6%)	674 (25.3%)	396 (25.1%)	
	moderate	308 (6.0%)	43 (4.6%)	141(5.3%)	124 (7.9%)	
	high	27 (0.5%)	4 (0.4%)	17 (0.6%)	6 (0.4%)	<0.001 *
Capsular fibrosis	with	1814 (35.1%)	273 (29.4%)	929 (34.8%)	612 (38.8%)	
	without	3359 (64.9%)	645 (70.6%)	1739 (65.2%)	966 (61.2%)	<0.001 *
Calcification	with	2376 (45.9%)	357 (38.5%)	1230 (46.1%)	789 (50.0%)	
	without	2797 (54.1%)	570 (61.5%)	1438 (53.9%)	789 (50.0%)	<0.001 *
Lipomatous atrophy	≤30%	3464 (67.0%)	589 (63.5%)	1852 (69.4%)	1023 (64.8%)	
	31–60%	678 (13.1%)	111 (12.0%)	323 (12.1%)	244 (15.5%)	
	≥61%	1031 (19.9%)	227 (24.5%)	493 (18.5%)	311 (19.7%)	<0.001 *

**Table 3:** Studies on age-related changes in human LNs.

Study	Patients	Age	LN (n)	Localization	Tumor/Non-Tumor	Reported Age-Related Morphological Changes
Hadamitzky	41	17–98	41	nm	non-tumor	Loss of lymphocytes/high endothelial venules, degree of fibrosis and lipomatosis
Pan	7	81–98	161	head and neck	nm	transparent LN
Erofeeva	10	75–90	nm	carinal/lower tracheo-bronchial	non-tumor	Thick edematous capsule, fibrous trabeculae located between the capsule and the node parenchyma, enlarged blood vessels, and a suppression of lymphocytopoietic function
Erofeeva	nm	77 +/− 6.78	13	mesenteric	non-tumor	Thickening of the capsule and trabeculae, proliferation of connective tissue around blood vessels and in the lymphoid parenchyma, lipomatosis of both cortical and medulla, decrease in the cell density in all structural components
Murakami	42	68–95	419	cervical, axillary, thoraxix, abdominal, pelvic, inguinal	non-tumor	Gaps in the cortex, fatty tissue infiltration, hyalinization
Tsakraklides	487	<16–>60	2250	axillary	tumor/non-tumor	Hyaline deposits increase with age
Denz	150	nm	300	deep cervical, inguinal, bronchial, mesenteric, axillary	tumor/non-tumor	Differences in LN function and anatomical position, retrogression of lymphoid tissue commences at puberty, lipomatous atrophy depends on nutritional state, collagenous and fibrous change
Luscieti	nm	0–90	243	cervical, axillar, cubital, inguinal, popliteal, mesenteric	non-tumor	Differences in anatomical position in size, lympatic tissue, and lipomatous atrophy
Taniguchi	27 + 12	72–95	519	cervical, axillar, mediastinal, abdominal, pelvic	non-tumor	Differences in hyalinization (mediastinal vs. pelvic type)
Sato	27	72–95	205	abdominal, pelvic	non-tumor	Region-specific histological heterogeneity with gap formation and thickend trabeculae

nm = not mentioned.

## Data Availability

The datasets used and/or analyzed during the current study are available from the corresponding author on reasonable request.

## Abbreviations

LN—lymph node; PLND—pelvic lymph node dissection; BC—bladder cancer; PC—prostate cancer; PSMA—prostate-specific membrane antigen; FRC—fibroblastic reticular cells.
